# Left ventricular global longitudinal strain predicts elevated cardiac pressures and poor clinical outcomes in patients with non-ischemic dilated cardiomyopathy

**DOI:** 10.1186/s12947-021-00254-1

**Published:** 2021-06-05

**Authors:** Ieva Kažukauskienė, Giedrė Balčiūnaitė, Vaida Baltrūnienė, Jelena Čelutkienė, Vytė Valerija Maneikienė, Sigitas Čibiras, Kęstutis Ručinskas, Virginija Grabauskienė

**Affiliations:** 1 Department of Pathology, Forensic Medicine and Pharmacology, Institute of Biomedical Science, Faculty of Medicine, Vilnius University, M. K. Čiurlionio 21, 03101 Vilnius, Lithuania; 2 Clinic of Cardiac and Vascular Diseases, Institute of Clinical Medicine, Faculty of Medicine, Vilnius University, M. K. Čiurlionio 21, 03101 Vilnius, Lithuania; 3 Center of Cardiology and Angiology, Vilnius University Hospital Santaros Klinikos, Santariškių 2, 08661 Vilnius, Lithuania

**Keywords:** Non-ischemic dilated cardiomyopathy, Global longitudinal strain, Strain-based index, Prognosis, Invasive hemodynamics, Heart failure

## Abstract

**Background:**

Risk stratification in patients with non-ischemic dilated cardiomyopathy (NI-DCM) is essential to treatment planning. Global longitudinal strain (GLS) predicts poor prognosis in various cardiac diseases, but it has not been evaluated in a cohort of exclusively NI-DCM. Although deformation parameters have been shown to reflect diastolic function, their association with other hemodynamic parameters needs further elucidation. We aimed to evaluate the association between GLS and E/GLS and invasive hemodynamic parameters and assess the prognostic value of GLS and E/GLS in a prospective well-defined pure NI-DCM cohort.

**Methods and results:**

Forty-one patients with NI-DCM were enrolled in the study. They underwent a standard diagnostic workup, including transthoracic echocardiography and right heart catheterization. During a five-year follow-up, 20 (49%) patients reached the composite outcome measure: LV assist device implantation, heart transplantation, or cardiovascular death.

Pulmonary capillary wedge pressure (PCWP), mean pulmonary artery pressure, pulmonary vascular resistance (PVR) correlated with GLS and E/GLS (*p* < 0.05). ROC analysis revealed that GLS and E/GLS could identify elevated PCWP (≥ 15 mmHg) and PVR (> 3 Wood units). Survival analysis showed GLS and E/GLS to be associated with short- and long-term adverse cardiac events (*p* < 0.05). GLS values above thresholds of –5.34% and -5.96% indicated 18- and 12-fold higher risk of poor clinical outcomes at one and five years, respectively. Multivariate Cox regression analysis revealed that GLS is an independent long-term outcome predictor.

**Conclusion:**

GLS and E/GLS correlate with invasive hemodynamics parameters and identify patients with elevated PCWP and high PVR. GLS and E/GLS predict short- and long-term adverse cardiac events in patients with NI-DCM. Worsening GLS is associated with incremental risk of long-term adverse cardiac events and might be used to identify high-risk patients.

## Introduction

Non-ischemic dilated cardiomyopathy (NI-DCM)—one of the main causes of heart failure—eventually leads to a high need for device therapy and heart transplantation. Heart failure, due to NI-DCM, accounts for 51–64% of all heart transplantations in the age group of 18–59 [1]. There is a need for reliable markers for identification of high-risk patients because they require close follow-up and timely decisions regarding advanced treatments.

Left ventricular (LV) global longitudinal strain (GLS) is a well-validated, easily performed echocardiographic parameter for evaluating myocardial deformation. GLS predicts poor prognosis in various cardiac diseases, including ischemic heart disease [2, 3] and heart failure with reduced ejection fraction (HFrEF) [4–6]. However, studies investigating mixed HFrEF populations have also included patients with ischemic heart disease (usually half of the cohort), who have a worse prognosis than patients with non-ischemic heart failure [7, 8]. Therefore, GLS prognostic significance in the HFrEF population has been confounded by the inclusion of ischemic heart failure patients. The predictive value of GLS has not been evaluated in patients with purely NI-DCM.

Evaluation of hemodynamic parameters is essential in prognostication and heart failure management, including device therapy and heart transplantation [9, 10]. Echocardiographic assessment is the main non-invasive diagnostic modality for the estimation of hemodynamic parameters. However, the relationship between various conventional echocardiographic parameters and invasively assessed pressures varies significantly in different studies [11–15], with each parameter having limitations. Thus, there is a need for new non-invasive parameters to judge about cardiac pressures. Several studies have proposed various diastolic strain-based indices that correlate with LV filling pressures [16–21], but variation in the parameters and technical challenges limit their use in clinical practice. Recently, Hayashi et al. [22] proposed a strain-based index of mitral E velocity ratio to GLS (E/GLS). The idea of the E/GLS was developed on the conventional echocardiographic parameter E/e’ – a ratio of early-diastolic LV inflow velocity (E) to early-diastolic mitral annular velocity (e´). It is known that e’ reflects longitudinal LV wall’s expansion rate, but it is angle-dependent, affected by heart translation motion. In comparison, GLS reflects longitudinal deformation of the whole ventricle and does not have the above-mentioned e’ limitations. This new index is strongly associated with LV mean diastolic pressure. Their findings encouraged further research in the relationship between invasive hemodynamic parameters and myocardial deformation measures.

Here, we aimed to evaluate the association of both GLS and E/GLS with invasive hemodynamic parameters; to evaluate the prognostic value of GLS and E/GLS for adverse cardiac events in a well-defined cohort of NI-DCM patients.

## Methods

### Study population and protocol

We enrolled 57 patients with suspected NI-DCM who were admitted to the university hospital for diagnostic evaluation between January 2010 and December 2013. Inclusion criteria were heart failure signs and symptoms, accompanied by echocardiographic evidence of LV dilatation and reduced (≤ 45%) LV ejection fraction (LVEF). The study’s primary aim was to identify etiopathogenetic factors—cardiotropic viruses and myocardial inflammation—of NI-DCM by evaluating various biomarkers in serum and endomyocardial biopsies [23, 24]. Forty-one patients had echocardiographic images of sufficient quality for further two-dimensional myocardial deformation analysis. These patients comprise the cohort of the present echocardiographic sub-study.

Exclusion criteria were: 1) significant coronary artery disease, defined as at least 50% proximal stenosis of a coronary artery, or a history of myocardial infarction; 2) other causes of heart failure, such as primary valvular or heart muscle disease, hypertensive heart disease, endocrine disease, advanced chronic kidney disease, drug or alcohol abuse; or 3) acute myocarditis (onset in the previous three months), or acute myocardial infarction as suspected by clinical presentation or diagnostic tests.

Study patients underwent a clinical evaluation and routine laboratory tests, including complete blood count, creatinine, and high-sensitivity C-reactive protein. Additionally, high-sensitivity troponin T (hs-TnT) was measured in serum using an Elecsys 2010 analyzer (Roche Diagnostics, Indianapolis, Indiana) and B-type natriuretic peptide (BNP) using ARCHITECT i analyzer (Abbott, Illinois, USA). Laboratory tests were performed in the laboratory of our university hospital, which is accredited according to the international standard EN/ISO-IEC 17,025.

Patients underwent transthoracic echocardiography on the same day or day before interventional procedures: coronary angiography and right heart catheterization. All patients were treated according to the guidelines of the European Society of Cardiology [25] and provided informed consent.

### Echocardiography

Echocardiographic evaluation was carried out using commercially available ultrasound machines (GE Vivid 7 or 9) with a 2.5-MHz probe. Images were digitally stored and analyzed offline using EchoPAC version PCBT08. We used a routine protocol of our laboratory for conventional M-mode, two-dimensional, Doppler, and tissue Doppler echocardiographic measurements [26]. LV end-diastolic (LVEDD) diameter was measured from the parasternal long-axis view and indexed to the body surface area. LV end-systolic and end-diastolic volumes were measured, and LVEF was calculated by the Simpson biplane method. Left atrium volume was measured by a biplane area-length method from the apical four- and two-chamber views and indexed to the body surface area. Mitral E and A peak velocity and deceleration time were measured, and the ratio of early-diastolic LV inflow velocity to atrial-systolic velocity (E/A) calculated. The average tissue Doppler-derived early diastolic mitral annular velocity (e’) was obtained from the mitral annulus’ septal and lateral sides. The average ratio of early-diastolic LV inflow velocity to early-diastolic mitral annular velocity (E/e´) was calculated. Mitral regurgitation and tricuspid regurgitation severity, as well as right ventricular function, were assessed visually.

### Myocardial deformation analysis by two-dimensional speckle tracking echocardiography

Echocardiographic images were acquired at 50–70 frames/s (with individual adjustment) for LV GLS analysis. A digital loop was acquired from three apical views (four-, two- and three-chamber views). After the manual cardiac cycle selection, the LV endocardial border was manually traced at the end-systolic frame (aortic valve closure was used for the end-systole timing). The investigator visually assessed the detected region of interest (ROI) and, if necessary, manually modified the ROI to ensure accurate tracking of the speckles. In the case of inaccurate speckle tracking, ROI was readjusted. We calculated the GLS by averaging the mean values of all valid segments. We also calculated a strain-based index, i.e., the ratio of early-diastolic LV inflow velocity (E) to GLS (E/GLS).

### Right heart catheterization

A Swan-Ganz catheter was inserted using a femoral approach in a supine position. The zero reference level of fluid-filled transducers was set at the mid-axillary line. Right atrium pressure, mean pulmonary artery pressure (mPAP), and pulmonary capillary wedge pressure (PCWP) were obtained. Wedge position in the PCWP measurement was confirmed by fluoroscopy, waveform changes, and arterial saturation ≥ 95%. Cardiac output (CO) was measured using Fick’s method and calculated by an equation: CO (l/min) = oxygen consumption (ml/min) / ((aorta SaO_2_ – pulmonary artery SvO_2_) x hemoglobin × 1.34). Only oxygen consumption (VO_2_) was estimated indirectly using VO_2_ nomograms based on age, weight and sex. Mixed venous blood was sampled for oximetry from the pulmonary artery, arterial blood – from the aorta. We calculated cardiac index (CO indexed to the body surface area), and pulmonary vascular resistance (PVR) ((mean PA pressure − PA wedge pressure)/CO) [27]. Each pressure measurement was recorded over a brief breath-hold at the end of expiration and was averaged over three consecutive cardiac cycles via computerized analysis [28]. On the basis of literature values, we used the following cut-offs for identifying elevated cardiac pressures: PCWP > 15 mmHg, mPAP, > 20 mmHg, and PVR > 3 Wood units [29].

### Follow-up

Patients were followed up for five years after enrollment in the study. The clinical outcome measure was a composite endpoint of LV assist device implantation, heart transplantation, or cardiovascular death. The time of the first event was included in the analysis. Adverse cardiac events were confirmed by medical records, national death registry records, or telephone interviews with the patients’ families.

### Statistical analysis

Data analysis was performed using the R studio package (4.0.3 version). A p-value of < 0.05 was considered statistically significant. The Shapiro–Wilk statistic tested continuous variables for normal distribution. Normally distributed continuous variables were expressed as the mean ± standard deviation. Other continuous variables were expressed as the median (25th percentile, 75th percentile), and categorical data as counts and percentages. Continuous variables were compared by Student’s independent t-test when normally distributed or by the Mann–Whitney-U test when non-normally distributed. Comparisons of categorical variables between the groups were made using the chi-square test or Fisher’s exact test if expected values were < 5. The association between echocardiographic and hemodynamic parameters was assessed using Spearman correlation.

The receiver operating characteristic (ROC) curve was used to estimate how well the echocardiographic parameters identified elevated hemodynamic parameters, predicted composite outcome measures, and identified the optimal cut-off value for the prediction. Differences between areas under the curve (AUC) were tested using the bootstrap method. Kaplan–Meier analysis was used to compare the cumulative survival rates between the two groups of NI-DCM patients stratified by the GLS, E/GLS or LVEF cut-off values. The log-rank statistic was used to evaluate the statistical significance of differences between the curves. Cox proportional hazards regression analysis was performed to evaluate which parameters were associated with poor one-year (short-term) and five-year (long-term) composite outcomes. We performed univariate Cox regression analysis for all baseline variables. The variables that were significant predictors in univariate analysis (*p* < 0.05) were enrolled in multivariate Cox regression analysis, which was performed using the stepwise backward elimination method.

## Results

### Baseline characteristics of the study population

The study included 41 patients with NI-DCM. The mean age was 47.0 ± 11.6 years and 33 (80%) patients were male. The majority of the patients were in the NYHA III-IV functional class. Patients had elevated PCWP (21 ± 8 mmHg), elevated mPAP (30 ± 12 mmHg), and low cardiac index (2.3 ± 0.7 l/min/m2) (Table 1). The mean LVEDD was 6.8 ± 0.8 cm, with a mean LVEF of 27.0 ± 9.1%. All patients had an impaired GLS (-8.1 ± 3.7%) (Table 2).

**Table 1:** Baseline characteristics of the study population, stratified by outcome

Variables	Total	Event-free group (*n* = 21)	Adverse cardiac event group (*n* = 20)	*p*-value
**Clinical characteristics**				
Age, years	47.0 ± 11.64	48.8 ± 10.0	45.2 ± 13.2	0.32
Male gender, n (%)	33 (80)	18 (86)	15 (75)	0.42
NYHA III-IV class, n (%)	36 (88)	17 (81)	19 (95)	0.34
Systolic BP, mm Hg	114 (104, 130)	120 (110, 130)	110 (103, 120)	0.09
Diastolic BP, mm Hg	80 (70, 80)	80 (70, 80)	73 (69, 80)	0.21
**Concomitant cardiac medication/**				
ACE-I/ARB, n (%)	31 (76)	17 (81)	14 (70)	0.48
Beta-blocker, n (%)	39 (95)	20 (95)	19 (95)	1
MRA, n (%)	37 (90)	19 (95)	18 (86)	0.6
Loop diuretics, n (%)	38 (93)	19 (91)	19 (95)	1
**Biomarkers**				
eGFR, ml/min/1.73 m^2^	83 (71, 102)	83 (73, 100)	86 (71, 103)	0.82
BNP, ng/l	809 (79, 1523)	300 (47, 851)	1294 (506. 2920)	< 0.01
Troponin T, pg/ml	29.1 (17.3, 46.5)	23.6 (9.4, 41.7)	33 (25.2, 65.2)	0.09
**Hemodynamic measurements**				
mPAP, mmHg	28 (21, 38)	23 (21, 34)	33 (27, 40)	0.09
Elevated mPAP (> 20 mmHg), n (%)	33 (80)	17 (81)	16 (80)	1
PCWP, mmHg	19 (15, 27)	17 (14, 22)	22 (16, 33)	0.18
Elevated PCWP (> 15 mmHg), n (%)	27 (66)	12 (57)	15 (75)	0.23
PVR, Wood units	1.9 (1.2, 3.1)	1.6 (1, 2.4)	2.5 (1.8, 3.5)	< 0.05
Elevated PVR (≥ 3 Wood units), n (%)	10 (25)	3 (15)	7 (35)	0.14
Cardiac index, l/min/m^2^	2.3 ± 0.7	2.4 ± 0.6	2.2 ± 0.8	0.49

Values are expressed as: mean ± SD, median (25th percentile, 75th percentile) or n (%)

*ACE-I* Angiotensin-converting enzyme inhibitor, *ARB* Angiotensin II receptor blocker, *BNP* B type natriuretic peptide, *BP* Blood pressure, *eGFR* Estimated glomerular filtration rate, *mPAP* mean pulmonary arterial pressure, *MRA* Mineralocorticoid receptor antagonist, *NYHA* New York Heart Association, *PCWP* pulmonary capillary wedge pressure, *PVR* pulmonary vascular resistance

**Table 2:** Echocardiographic characteristics of the study population, stratified by outcome

Variables	Total	Event-free group (*n* = 21)	Adverse cardiac event group (*n* = 20)	*p*-value
LVEF, %	25 (20, 34)	30 (25, 35)	21 (19, 23)	< 0.01
LV GLS, %	-8.1 ± 3.72	-9.9 ± 2.8	-6.2 ± 3.7	< 0.001
E/GLS [× 10^2^], cm/s	-8.9 (-18.0, -6.3)	-8.1 (-9.8, -5.8)	-14.8 (-25.6, -8.6)	< 0.01
LVEDD, cm	6.8 ± 0.8	6.6 ± 0.7	7.1 ± 0.9	0.06
LAVi, ml/m^2^	66 (50, 77)	55 (53, 76)	73 (46, 81)	0.63
Mitral DT, ms	164 (127, 194)	145 (111, 187)	142 (98, 187)	0.29
Mitral E/A	2.1 (0.9, 2.9)	2 (0.8, 2.7)	2.4 (1.2, 3.3)	0.44
Average E/e’	14.2 (12.3, 15.6)	13.7 (10.9, 15.1)	15.0 (13.1, 17.4)	0.13
Functional mitral regurgitation ≥ moderate, n (%)	24 (58)	11 (52)	13 (65)	0.41
Functional tricuspid regurgitation ≥ moderate, n (%)	16 (39)	6 (29)	10 (50)	0.16
RV end-diastolic diameter, cm	3.3 (3, 3.6)	3.2 (3.0, 3.5)	3.5 (3.1, 3.7)	0.08
Severely impaired RV systolic function, n (%)	13 (32)	5 (24)	8 (40)	0.27
TR systolic jet velocity (m/s)	2.7 (2.5, 3.2)	2.5 (2.4, 2.7)	2.8 (2.5, 3.0)	0.16

Values are expressed as: mean ± SD, median (25th percentile, 75th percentile) or n (%)

*DT* deceleration time, *E/A* ratio of early-diastolic LV inflow velocity (E) to atrial-systolic velocity (A), *E/e’* ratio of early-diastolic LV inflow velocity (E) to early-diastolic mitral annular velocity (e´), *E/GLS* ratio of early-diastolic LV inflow velocity (E) to global longitudinal strain (GLS), *GLS* global longitudinal strain, *LAVi* left atrial indexed volume, *LVEDD* left ventricular end-diastolic diameter, *LVEF* left ventricular ejection fraction, *TR* tricuspid regurgitation

During the five-year follow-up, twenty (49%) patients experienced at least one adverse cardiac event: 6 patients underwent LV assist device implantation, 5 had heart transplants, and 9 died. Those who experienced adverse cardiac event had more unfavorable baseline characteristics, such as higher serum B-type natriuretic peptide levels and higher PVR (Table 1). Echocardiographic baseline characteristics, such as LVEF, GLS and E/GLS were more unfavorable in adverse cardiac event groups (Table 2).

### Association between GLS, E/GLS and invasively measured cardiac pressures

**Fig. 1:**
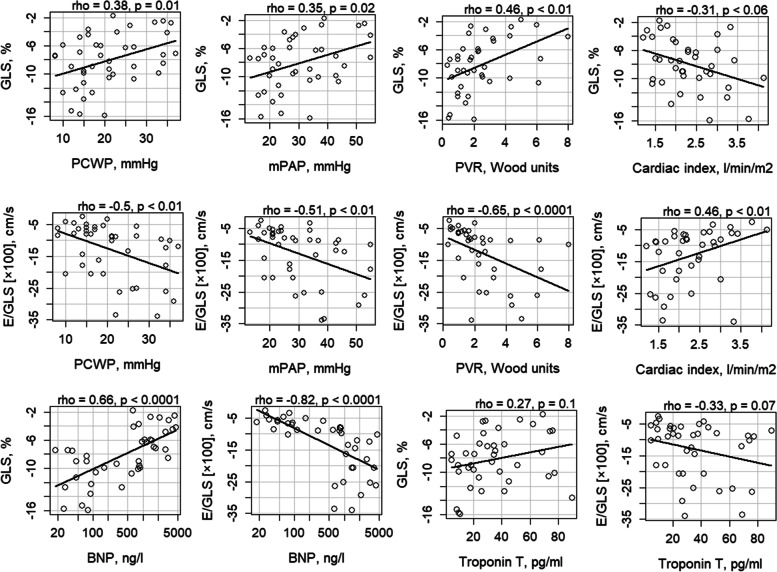
Correlations between myocardial deformation parameters and invasive hemodynamic measures, and serum biomarkers. BNP – B type natriuretic peptide; E/GLS – ratio of early-diastolic LV inflow velocity (E) to global longitudinal strain (GLS); GLS – global longitudinal strain; LVEF – left ventricle ejection fraction; mPAP – mean pulmonary arterial pressure; PCWP – pulmonary capillary wedge pressure; PVR – pulmonary vascular resistance

We evaluated the association between GLS, E/GLS and other conventional echocardiographic parameters with invasive cardiac pressures and cardiac index. GLS as well as average E/e’, TR velocity, LAVi and LVEF significantly correlated with cardiac pressures, while E/GLS correlated with cardiac pressures even stronger than GLS. E/GLS correlated with all cardiac pressures and cardiac index. The strongest correlation was between PVR and E/GLS (Table 3). Correlations between myocardial deformation parameters and invasive hemodynamic measurements are also plotted in Fig. 1. Additionally, we evaluated correlations between GLS, E/GLS and serum biomarkers. Both GLS and E/GLS strongly correlated with BNP, but not with troponin T. The strongest correlation was between E/GLS and BNP (Fig. 1).

**Table 3:** Correlations between echocardiographic parameters and invasive hemodynamic ones

	**PCWP, mmHg**		**mPAP, mmHg**		**PVR, Wood units**		**Cardiac index, l/min/m**^**2**^	
	*r*	*p*	*r*	*P*	*r*	*p*	*r*	*p*
**DT, ms**	-0,13	0.42	-0.17	0.28	**-0.4**	0.01	0.21	0.21
**E/A**	0.22	0.21	**0.38**	0.03	**0.62**	< 0.0001	**-0.55**	< 0.01
**Average E/e’**	**0.37**	0.02	**0.36**	0.02	**0.31**	0.048	-0.02	0.92
**TR velocity, m/s**	**0.54**	< 0.001	**0.59**	< 0.0001	**0.5**	< 0.001	-0.19	0.24
**LAVi, ml/m**^**2**^	**0.40**	< 0.01	**0.47**	< 0.01	**0.6**	< 0.0001	**-0.45**	< 0.01
**LVEF, %**	**-0.38**	0.01	**-0.41**	< 0.01	**-0.45**	< 0.01	0.26	0.11
**GLS, %**	**0.38**	0.01	**0.35**	0.02	**0.46**	< 0.01	-0.31	0.06
**E/GLS, [× 10**^**2**^**] cm/s**	**-0.5**	< 0.01	**-0.51**	< 0.01	**-0.65**	< 0.0001	**0.46**	< 0.01

The bold font character means statistically significance (*p* < 0.05)

*DT* deceleration time, *E/A* ratio of early-diastolic LV inflow velocity (E) to atrial-systolic velocity (A), *E/e’* ratio of early-diastolic LV inflow velocity (E) to early-diastolic mitral annular velocity (e´), *E/GLS* ratio of early-diastolic LV inflow velocity (E) to global longitudinal strain (GLS), *GLS* global longitudinal strain, *LAVi* left atrium indexed volume, *LVEF* left ventricular ejection fraction, *PCWP* pulmonary capillary wedge pressure, *mPAP* mean pulmonary arterial pressure, *PVR* pulmonary vascular resistance, *TR* tricuspid regurgitation

We then tested echocardiographic parameters’ ability to identify patients with elevated cardiac pressures. ROC analysis revealed that both GLS and E/GLS were good predictors of PCWP ≥ 15 mmHg and PVR > 3 Wood units, but not mPAP > 20 mmHg (Table 4). What stands out in Table 4 is that AUC was significant for GLS and E/GLS, but not for other echocardiographic parameters, except E/A, which predicted high PVR, and LAVi, which predicted elevated mPAP and high PVR.

**Table 4:** ROC analysis for identifying elevated hemodynamic parameters by echocardiographic markers

	**AUC (95% CI)**		
	**PAWP ≥ 15 mmHg**	**mPAP > 20 mmHg**	**PVR > 3 Wood units**
**DT, ms**	0.61 (0.42–0.79)	0.60 (0.37–0.81)	0.68 (0.49–0.86)
**E/A**	0.68 (0.49–0.86)	0.69 (0.45–0.90)	**0.85 (0.68–0.98)**
**Average E/e’**	0.62 (0.41–0.80)	0.53 (0.29–0.76)	0.67 (0.46–0.85)
**TR velocity, m/s**	0.68 (0.49–0.85)	0.66 (0.42–0.90)	0.51 (0.23–0.78)
**LAVi, ml/m**^**2**^	0.68 (0.50–0.85)	**0.78 (0.60–0.95)**	**0.76 (0.57–0.92)**
**LVEF, %**	0.65 (0.43–0.83)	0.63 (0.41–0.85)	0.66 (0.47–0.84)
**GLS, %**	**0.74 (0.57–0.88)**	0.65 (0.45–0.86)	**0.78 (0.60–0.95)**
**E/GLS, [× 10**^**2**^**] cm/s**	**0.76 (0.59–0.91)**	0.72 (0.49–0.92)	**0.84 (0.68–0.96)**

The bold font character means statistically significance (*p* < 0.05)

*AUC* area under the curve, *95% CI* 95% confidence interval, *DT* deceleration time, *E/A* ratio of early-diastolic LV inflow velocity (E) to atrial-systolic velocity (A), *E/e’* ratio of early-diastolic LV inflow velocity (E) to early-diastolic mitral annular velocity (e´), *E/GLS* ratio of early-diastolic LV inflow velocity (E) to global longitudinal strain (GLS), *GLS* global longitudinal strain, *LAVi* left atrium indexed volume, *PCWP* pulmonary capillary wedge pressure, *mPAP* mean pulmonary arterial pressure, *PVR* pulmonary vascular resistance, *TR* tricuspid regurgitation

### Prognostic utility of GLS and E/GLS

**Fig. 2:**
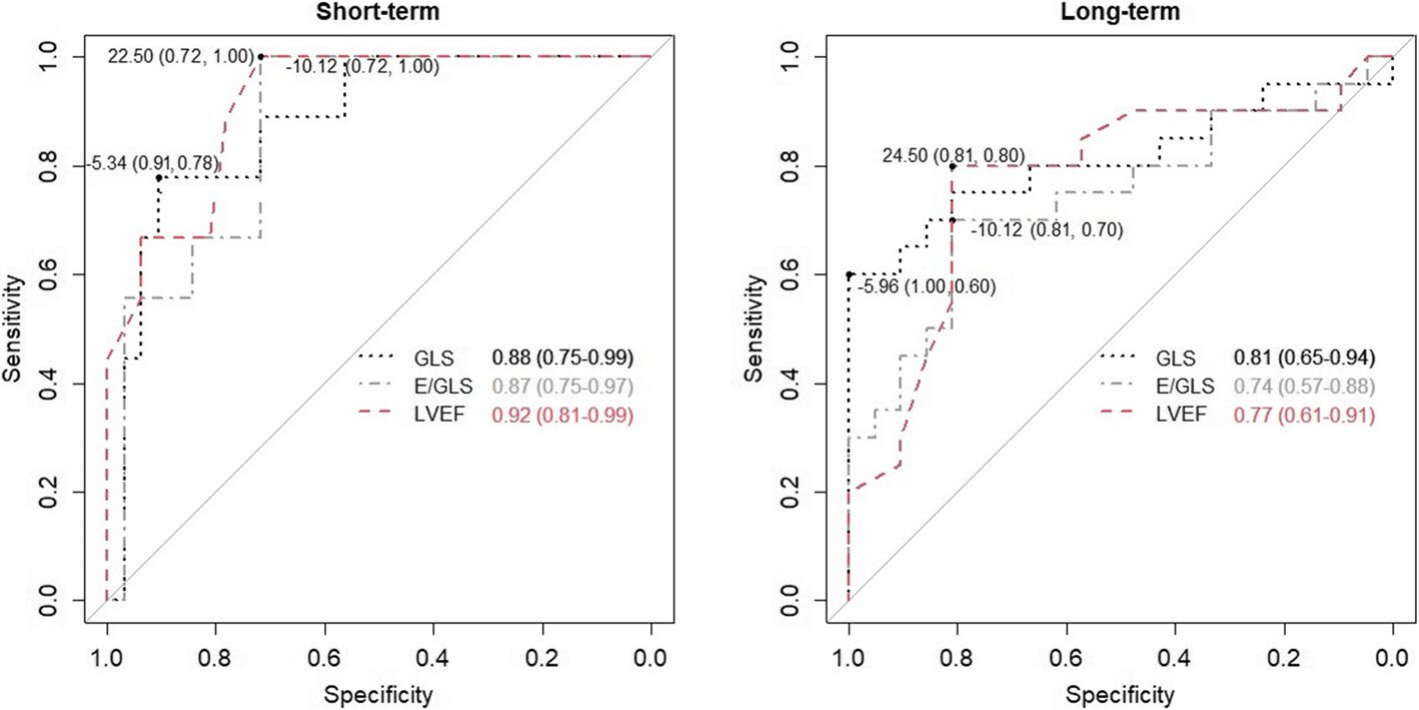
ROC analysis of GLS, E/GLS and LVEF identified high-risk patients for an adverse cardiac event. E/GLS – ratio of early-diastolic LV inflow velocity (E) to global longitudinal strain (GLS); GLS – global longitudinal strain; LVEF – left ventricle ejection fraction

We then used GLS, E/GLS and LVEF to identify high-risk patients in terms of adverse cardiac events. For comparison, we chose LVEF as the most widely used prognostic echocardiographic parameter in clinical practice. ROC analyses showed, that GLS, E/GLS and LVEF predicted poor clinical outcomes during the short- and long-term follow-ups (Fig. 2). The differences between the curves were not significant. Additionally, the analysis identified optimal cut-off values for the short- and long-term composite outcome measure: -5.34% (78% sensitivity, 91% specificity) and -5.96% (60% sensitivity, 100% specificity) for GLS, -10.12 cm/s (70 and 100% sensitivity, 72 and 81% specificity) for E/GLS, and 22.5% (100% sensitivity, 72% specificity) and -24.5% for LVEF (80% sensitivity, 81% specificity), respectively.

**Fig. 3:**
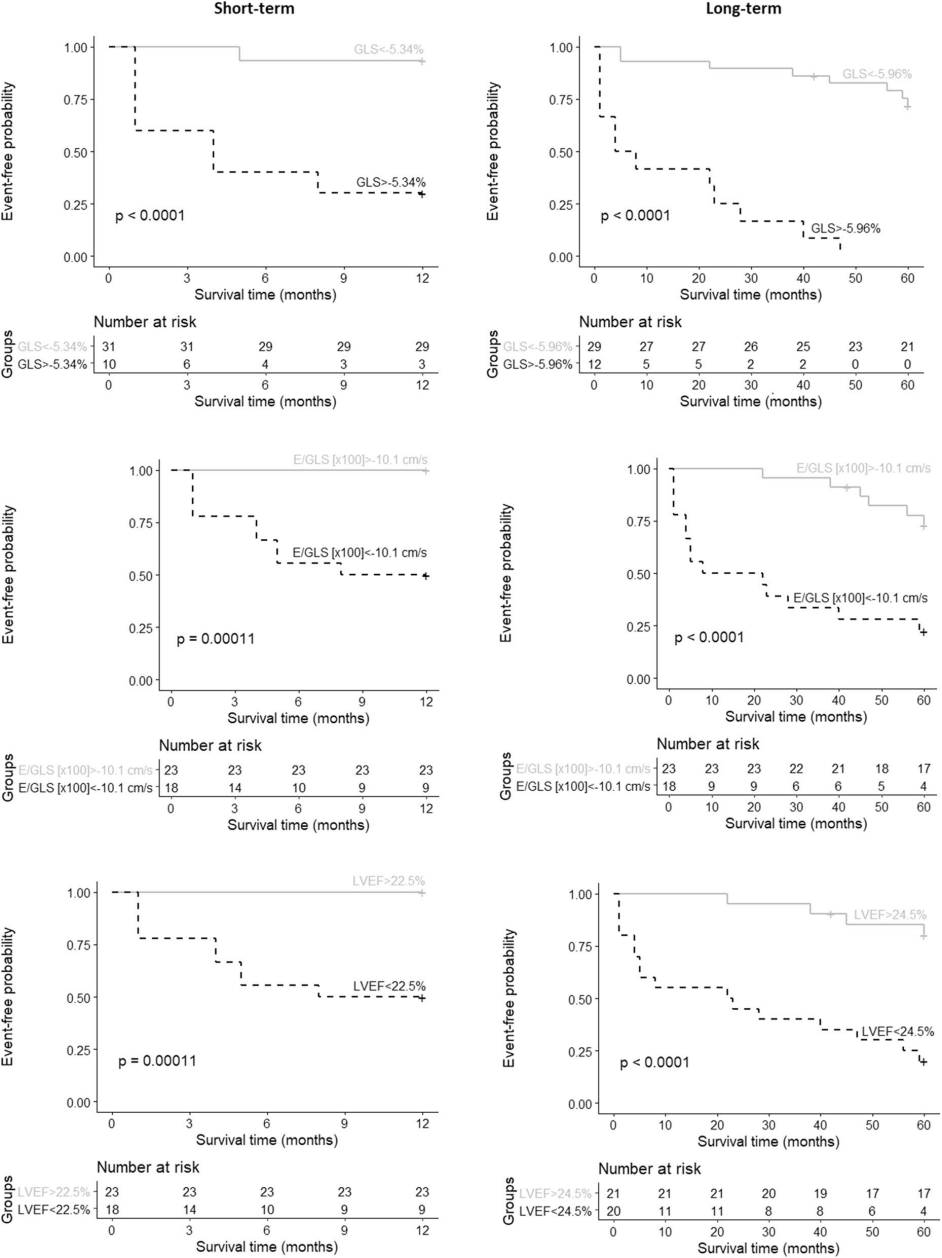
Survival curves stratified by GLS, E/GLS and LVEF during short- and long-term follow-ups. E/GLS – ratio of early-diastolic LV inflow velocity (E) to global longitudinal strain (GLS); GLS – global longitudinal strain; LVEF – left ventricle ejection fraction

To obtain additional information for risk stratification, we performed survival analysis with GLS, E/GLS and LVEF. The survival curve estimations demonstrated that patients with GLS values above the cut-off or E/GLS and LVEF below it had significantly lower event-free survival rates during both short- and long-term follow-up (*p* < 0.001) (Fig. 3). The graph shows that more than half of the patients with GLS above the cut-off value experienced adverse cardiac events during the first year of follow-up. In addition, univariate Cox analysis demonstrated that GLS values above cut-offs indicated 18- (HR 18.52; 95% CI 3.79–90.41, *p* < 0.01) and 12-fold (HR 12.47; 95% CI 4.6–33.82, < 0.0001) higher risk of poor clinical outcomes at one and five years, respectively.

**Fig. 4:**
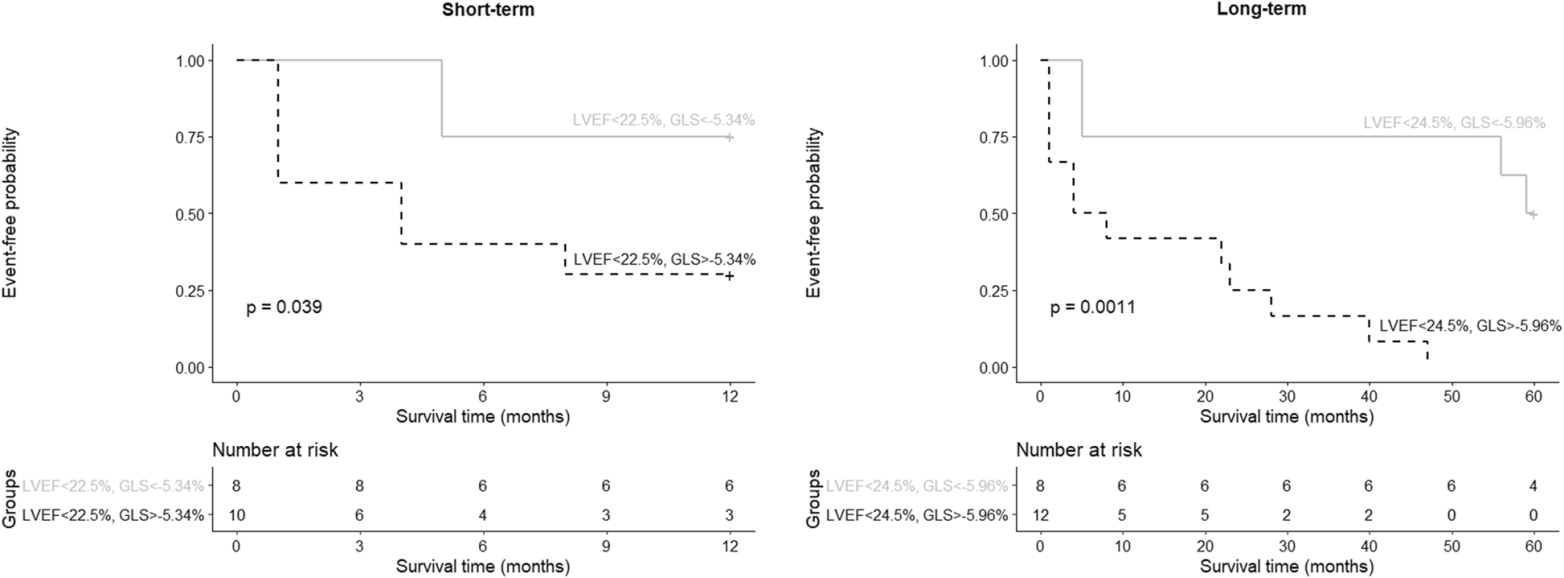
Composite adverse cardiac events probability according to GLS within severely reduced LVEF patients. GLS – global longitudinal strain; LVEF – left ventricle ejection fraction

Given that LVEF is a well-established prognostic marker, a subgroup survival analysis was performed to evaluate whether GLS has an additional predictive value in patients stratified by LVEF. In patients with LVEF values above the cut-off, GLS did not stratify the risk, while all patients had GLS values below the cut-off. However, for patients with LVEF below the cut-off value, the risk was further stratified by GLS (Fig. 4). Patients with GLS above the cut-off value (short-term GLS > -5.34% or long-term > -5.96%) had significantly increased events rates within the severely reduced LVEF group.

We then performed univariate Cox proportional-hazards model analysis to evaluate the prognostic significance of GLS and E/GLS as continuous variables. All baseline variables from Tables 1 and 2 were enrolled in univariate Cox regression analysis. The analysis showed that arterial blood pressure, severe right ventricle systolic dysfunction and cardiac index were associated only with short-term clinical outcomes (Table 5). While GLS, E/GLS, LVEF, BNP, Troponin T cardiac pressures and right ventricle enlargement were associated with adverse cardiac events during short- and long-term follow-up (Tables 5 and 6). GLS increase by 1% was associated with 55% and 41% higher risk of adverse cardiac events during short- and long-term follow-up, respectively.

**Table 5:** Results of Cox regression analysis for predictors of adverse cardiac events during short-term follow-up

	**Univariate**		**Multivariate**	
	**HR (95% CI)**	***p***	**HR (95% CI)**	***p***
Systolic BP, mm Hg	0.95 (0.9–0.99)	0.03		
Diastolic BP, mm Hg	0.93 (0.86–0.998)	0.04		
BNP, ng/l	1.001 (1–1.001)	< 0.0001	1.001 (1.0–1.0014)	< 0.01
Troponin T, pg/ml	1.004 (1–1.008)	0.03	1.007 (1.002–1.01)	< 0.01
mPAP, mmHg	1.09 (1.03–1.16)	< 0.01		
PCWP, mmHg	1.16 (1.06–1.28)	< 0.01		
PVR, Wood units	1.53 (1.13–2.08)	< 0.01		
Cardiac index, l/min/m^2^	0.16 (0.04–0.77)	0.02		
Severely impaired RV systolic function	5.29 (1.32–21.14)	0.02		
RV end-diastolic diameter, cm	2.44 (1.08–5.52)	0.03		
LVEF, %	0.8 (0.69–0.92)	< 0.01	0.84 (0.72–0.99)	0.04
LV GLS, %	1.55 (1.19–2.03)	< 0.001		
E/GLS [× 10^2^], cm/s	0.96 (0.93–0.995)	0.02		

*BNP* B type natriuretic peptide, *BP* Blood pressure, *CI* confidence interval, *E/GLS* ratio of early-diastolic LV inflow velocity (E) to global longitudinal strain (GLS), *GLS* global longitudinal strain, *HR* hazard ratio, *LVEF* left ventricular ejection fraction, *mPAP* mean pulmonary arterial pressure, *PCWP* pulmonary capillary wedge pressure, *PVR* pulmonary vascular resistance, *RV* right ventricle

**Table 6:** Results of Cox regression analysis for predictors of adverse cardiac events during long-term follow-up

	**Univariate**		**Multivariate**	
	**HR (95% CI)**	***p***	**HR (95% CI)**	***p***
BNP, ng/l	1.001 (1–1.001)	< 0.0001	1.001 (1.0 -1.001)	0.02
Troponin T, pg/ml	1.004 (1.001–1.008)	0.02	1.004 (1.0–1.009)	0.04
mPAP, mmHg	1.1 (1.04–1.16)	0.002		
PCWP, mmHg	1.18 (1.05–1.13)	0.007		
PVR, Wood units	1.28 (1.03–1.59)	0.03		
RV end-diastolic diameter, cm	2.45 (1.24–4.87)	0.01		
LVEF, %	0.89 (0.83–0.96)	< 0.01		
LV GLS, %	1.41 (1.18–1.68)	< 0.0001	1.25 (1.01–1.55)	0.04
E/GLS [× 10^2^], cm/s	0.96 (0.93–0.98)	< 0.001		

*BNP* B type natriuretic peptide, *CI* confidence interval, *E/GLS* ratio of early-diastolic LV inflow velocity (E) to global longitudinal strain (GLS), *GLS* global longitudinal strain, *HR* hazard ratio, *LVEF* left ventricular ejection fraction, *mPAP* mean pulmonary arterial pressure, *PCWP* pulmonary capillary wedge pressure, *PVR* pulmonary vascular resistance, *RV* right ventricle

The significant univariate predictors were enrolled in multivariate Cox regression analysis, which was performed using stepwise backward elimination method. GLS showed significant association with the occurrence of adverse cardiac events during long-term follow-up (adjusted HR 1.25 (95% CI 1.01–1.55); *p* = 0.04), even after adjusting for univariate outcome predictors (Table 6). However, E/GLS were not significantly associated with clinical outcomes after adjusting for other univariate predictors.

## Discussion

This study evaluates the association between myocardial deformation parameters and invasively assessed cardiac pressures and PVR as well as the prognostic value of both GLS and E/GLS for predicting clinical outcomes in NI-DCM patients. The main findings are that:

1. GLS and E/GLS correlate with PCWP, mPAP, PVR, and can predict elevated cardiac pressures
2. GLS and E/GLS are significantly associated with poor clinical outcome (both short- and long-term) in a well-defined NI-DCM cohort;
3. GLS > -5.34 and > -5.96% predicts adverse clinical events during one-year and five-year follow-ups, respectively. In the subgroup of patients with severely reduced LVEF, GLS values above the cut-off have an additional predictive value.

Echocardiographic evaluation of LV filling pressure is essential for HFrEF patients. Guidelines recommend a multiparametric echocardiographic approach for the evaluation [30]. Nevertheless, each parameter has limitations and may provide inconsistent results. In addition, their association with invasive LV filling pressures varies across studies [12, 13, 31]. For these reasons, there is a need for additional, non-invasive parameters for LV filling pressure evaluation. Recently, Hayashi et al. [22] in the study of 77 patients (39% had HFrEF) have demonstrated a correlation between GLS and time relaxation constant (tau). However, they did not evaluate the association between GLS and LV filling pressure. They have proposed a strain-based index E/GLS and demonstrated a correlation between E/GLS and LV mean diastolic pressure. Romano et al. evaluated 78 patients with various etiology of HFrEF. They have demonstrated that four-chamber longitudinal strain was a predictor of elevated PCWP [32]. In agreement with this study, we have also estimated PCWP as a surrogate parameter for LV filling pressure. PCWP approximates the left atrial pressure, which, in turn, approximates LV end-diastolic pressure in the absence of pulmonary vein and mitral valve stenosis. In our study, GLS significantly, albeit weakly, correlated with PCWP. Furthermore, PCWP correlated with strain-based index E/GLS, and the correlation was stronger than the one between GLS and PCWP. We also found that GLS and E/GLS identified patients with elevated PCWP. These associations were also supported by strong correlations between myocardial deformation parameters and BNP, which secretion increases as a response to myocardial wall stretch due to pressure or volume overload.

Elevated mPAP and increased PVR are the consequence of long-lasting abnormal LV filling pressure in our cohort. The backward transmission of elevated LV filling pressure might also explain GLS and E/GLS correlation not only with PCWP, but also with mPAP and PVR. E/GLS correlated more strongly with these parameters than did GLS. Interestingly, the strongest correlation was between PVR and E/GLS. The ROC curve analysis demonstrated that both E/GLS and GLS could identify patients with PVR > 3 Wood units. This PVR value differentiates two distinct hemodynamic phenotypes of post-capillary pulmonary hypertension: isolated post-capillary (≤ 3 Wood units) hypertension from combined post-capillary and pre-capillary pulmonary hypertension (> 3 Wood units). This distinction is essential for accurate prognostication and treatment decision-making [33].

Prior studies have demonstrated that GLS has a significant predictive value in heart failure patients [4–6, 34], in various cardiovascular pathologies [35–42], and even in the general population [43]. To our knowledge, this is the first study to investigate the predictive potential of GLS and E/GLS ratio in patients with chronic heart failure due to NI-DCM. Our study showed that both GLS and E/GLS are predictors of poor short- and long-term outcomes in a well-defined cohort of patients with NI-DCM. Furthermore, GLS remained a significant long-term predictor when added to a model with other prognostic parameters.

Guidelines define a GLS ≤ –20% as a normal value in healthy subjects [26]. A recent meta-analysis reports that a GLS > -16% indicates significant myocardial dysfunction [44]. There are no defined GLS cut-off values for risk estimation in different cardiac pathologies, although these would be useful for prognostication, management, and future studies. Motoki et al. [4] included 194 patients with various etiology chronic heart failure. They identified a GLS cut-off value of -6.95% to be a predictor of poor clinical outcomes during a five-year follow-up. We identified the cut-off value of GLS -5.96% of adverse cardiac events during a five-year follow-up. The worse GLS cut-off value in our study compared to theirs might be due to the severity of heart failure in our cohort (NYHA III-IV functional class 88% versus 39%) and a higher event rate (49% versus 40%). Sengelov et al. [5] included 1065 patients with various origin HFrEF and found a mortality rate of 16.7% during the median follow-up of 40 months. They identified a GLS cut-off value of -5.9% to be a useful predictor of increased mortality in patients with severely reduced LV systolic function (LVEF < 22%). Our study’s identified cut-off value is in line with their study, despite differences in heart failure etiology, follow-up duration, and event rate. It is important to note that our cut-off value had 100% specificity, supported by Kaplan–Meier analysis: all patients with GLS > -5.96% experienced adverse cardiac events before the end of follow-up. In contrast to previous studies, we evaluated not only long-term but also short-term clinical outcomes. Our study’s cut-off value (GLS > -5.34%) predicted short-term clinical outcome, with more than half of the patients experiencing cardiac events during the first year of follow-up. These cut-offs might be useful for identifying patients with advanced heart failure.

### Limitations and clinical implications

Our study’s main limitation is the small sample size, which might explain why we did not find any significant differences between AUCs of echocardiographic parameters in the ROC analysis. In addition, echocardiography was not performed simultaneously with a right heart catheterization. This might lead to differences in loading conditions, which in turn might reflect a weak to absent correlation between several echocardiographic and invasively measured parameters.

Despite the limitations, the study demonstrated that E/GLS might be an additional parameter for LV filling pressure assessment, while E/GLS correlated with PCWP stronger than most conventional echocardiographic parameters and GLS. E/GLS might improve LV diastolic function evaluation and might aid in clinical scenarios where conventional echocardiographic parameters’ usage is limited, i.e., mitral valve pathology, advanced heart failure. However, a larger study is needed to estimate in which clinical scenarios E/GLS would be beneficial.

To our best knowledge, none of the previous studies evaluated E/GLS prognostic value. However, GLS seems to be a superior predictor than E/GLS, as it was an independent predictor for long-term clinical outcomes and further stratified patients with severely reduced LV systolic function. The use of GLS to identify high-risk NI-DCM patients could lead to changes in follow-up intensity, the timing for device therapy, or prioritization on the heart transplantation list.

## Conclusion

GLS and E/GLS correlate with invasive hemodynamics parameters and identify patients with elevated PCWP and high PVR. GLS and E/GLS predict short- and long-term adverse cardiac events in patients with NI-DCM. Worsening GLS is associated with incremental risk of long-term adverse cardiac events and might be used to identify high-risk patients.

## Data Availability

The datasets during and/or analyzed during the current study available from the corresponding author on reasonable request.

## Abbreviations

ACE-I: Angiotensin-converting enzyme inhibitor
ARB: Angiotensin II receptor blocker
BNP: B type natriuretic peptide
BP: Blood pressure
DT: Deceleration time
E/A: Ratio of early-diastolic LV inflow velocity (E) to atrial-systolic velocity (A)
E/e’: Ratio of early-diastolic LV inflow velocity (E) to early-diastolic mitral annular velocity (e´)
E/GLS: Ratio of early-diastolic LV inflow velocity (E) to global longitudinal strain (GLS)
eGFR: Estimated glomerular filtration rate
GLS: Global longitudinal strain
LAVi: Left atrial indexed volume
LV: Left ventricle
LVEDD: Left ventricular end-diastolic diameter
LVEF: Left ventricular ejection fraction
mPAP: Mean pulmonary arterial pressure
MRA: Mineralocorticoid receptor antagonist
NYHA: New York Heart Association
NI-DCM: Non-ischemic dilated cardiomyopathy
PCWP: Pulmonary capillary wedge pressure
PVR: Pulmonary vascular resistance
TR: Tricuspid regurgitation
